# Predicting siRNA efficacy based on multiple selective siRNA representations and their combination at score level

**DOI:** 10.1038/srep44836

**Published:** 2017-03-20

**Authors:** Fei He, Ye Han, Jianting Gong, Jiazhi Song, Han Wang, Yanwen Li

**Affiliations:** 1 Northeast Normal University, School of Computer Science and Information Technology, Changchun, 130117, China; 2 Northeast Normal University, School of Environment, Changchun, 130117, China; 3 Northeast Normal University, Institute of Computational Biology, Changchun, 130117, China; 4 Jilin University, College of Computer Science and Technology, Changchun, 130012, China; 5 Jilin University, Key Laboratory of Symbolic Computation and Knowledge Engineering of Ministry of Education, Changchun, 130012, China

## Abstract

Small interfering RNAs (siRNAs) may induce to targeted gene knockdown, and the gene silencing effectiveness relies on the efficacy of the siRNA. Therefore, the task of this paper is to construct an effective siRNA prediction method. In our work, we try to describe siRNA from both quantitative and qualitative aspects. For quantitative analyses, we form four groups of effective features, including nucleotide frequencies, thermodynamic stability profile, thermodynamic of siRNA-mRNA interaction, and mRNA related features, as a new mixed representation, in which thermodynamic of siRNA-mRNA interaction is introduced to siRNA efficacy prediction for the first time to our best knowledge. And then an *F*-score based feature selection is employed to investigate the contribution of each feature and remove the weak relevant features. Meanwhile, we encode the siRNA sequence and existed empirical design rules as a qualitative siRNA representation. These two kinds of siRNA representations are combined to predict siRNA efficacy by supported Vector Regression (SVR) at score level. The experimental results indicate that our method may select the features with powerful discriminative ability and make the two kinds of siRNA representations work at full capacity. The prediction results also demonstrate that our method can outperform other popular siRNA efficacy prediction algorithms.

At 1998, Fire first introduced RNA interference (RNAi) mechanism, in which ribonuclease III enzyme Dicer is able to cleave a long double stranded RNA (dsRNA) duplex into small interfering RNAs (siRNAs) with 19 nucleotides (nt) sequences and 2 nt overhangs at the 3′ ends^1^. Then siRNAs bind to RNA-induced silencing complex (RISC), which may guide to the degradation of complementary targeted messenger RNA (mRNA) and gene knockdown. Due to its gene silencing function, RNAi has been considered a promising approach to help treat targeted diseases such as AIDS^2^, neurodegenerative diseases^3^, and cancer^4^. However, the gene silencing effectiveness of RNAi relies on the siRNA efficacy in targeting a specific gene. Thereby, an effective siRNA efficacy prediction method constitutes a huge challenge for selecting the most active siRNA.

In the early works, researchers depended on several sets of empirical rules from experimental data to select potent siRNA. The first rules proposed by Elbashir indicate that an efficient siRNA should have 19 nt sequence with 2 nt overhangs at the 3′ ends^5^. In addition, Scherer^6^ pointed out that the thermodynamic properties to target specific mRNAs need to be considered in siRNA design. Subsequently, many rational rules for designing active siRNA were found. For example, Reynolds analyzed 180 siRNA targeted the mRNA of two genes, and reported eight rule: (1) rich G/C content, (2) three or more A/U at positions 15–19 (3) absence of internal repeats, (4) position 19 with A, (5) position 3 with A, (6) position 10 with U, (7) position 19 without G/C, and (8) position 13 without G^7^. Ui-Tei studied 72 siRNAs targeted the mRNA of six genes, and suggested a serial criterions: (1) position 19 with A/T, (2) position 1 with G/C, (3) five or more T/A at positions 13–19, and (4) maximum of 9 nt long GC stretch^8^. Although these empirical rules are indispensable for siRNA design, the tools only using empirical rules can hardly reach our acceptable level. Because these rules are summarized from small scale dataset and focus on some specific gene only.

In recent years, several machine learning based algorithms emerged as siRNA data rates grew, especially after Huesken published a dataset consisting of 2431 siRNAs, whose knockdown efficacies and targeted mRNAs may experimentally observed^9^. These approaches involved more siRNAs and their characteristics, and exhibited more accuracy and reliability. For example, Huesken developed a tool named Biopredsi and applied artificial neural networks to predict siRNA efficacy^9^. Another tool ThermoComposition 21 combined position features and thermodynamic features to an artificial neural network model for further improving the prediction accuracy^10^. DSIR used basic sequence information and a simple linear model LASSO, which also achieved good performance^11^. In addition, two more models i-Score and Scales utilized linear regression models to perform art-of-the-state accuracy rates^12^,^13^. The five popular methods are considered as the best predictors^13^,^14^. These approaches almost employed heterogeneous siRNA features, including sequence composition and thermodynamic stability profile, and a regression or classification computational model to achieve great improvement compared with previous rule-based methods.

The machine learning methods suggested that the sequence and thermodynamic parameters of siRNA are strongly associated with the effectiveness of gene silencing. However, there are some shortcomings in existed methods: (1) several methods focused on characterizing siRNAs according to their sequences and profiles, but missed the application of the empirical rules; (2) few method took the thermodynamic of siRNA-mRNA interaction and mRNA-related features into consideration. And the literature^15^ demonstrated that the mRNA related feature might help predict siRNA efficacy; (3) Even though the tool siPred tried to combine the features together with the rules as input^16^, it neglected to deal with the data heterogeneity between the continuous and binary data, which may influence the accuracy of modeling a linear regression system.

Aiming at developing a more reliable and stable model to predict the siRNA knockdown efficacy, in our work, we focus on three main tasks: (1) constructing meaningful and rich representations of siRNAs, (2) selecting the most related features to represent siRNAs, (3) rationally combining these representations to build a improved siRNA efficacy predictor. In the first task, in order to objectively and comprehensively represent siRNA, we define two different types of representations to describe siRNA from both quantitative and qualitative analyses. The first description is a hybrid feature vector combining sequence frequencies, thermodynamic stability profile, thermodynamic of siRNA-mRNA interaction together with mRNA related information. All these features can be quantified, thus they are integrated into a continuum feature vector. For further analyzing the contribution of each component in the hybrid feature, we try to implement a feature selection algorithm to assess each component feature, and find out the optimal feature subset to remove the features with weak relevancy. In the second representation, we encode empirical siRNA design rules to qualitatively characterize siRNA. Subsequently, we consider the third task that fuses the two incompatible types of representations to level up the performance of prediction. Generally speaking, the common way to combine multiple types of features as a vector, also called feature fusion, is difficult to achieve improvement due to the heterogeneity and incompatibleness among different forms of features. Instead, score level fusion is more feasible and effective^17^. Therefore, we would like to address this combination problem by respectively using two Supported Vector Regression (SVR) models with different kernels to map the two heterogeneous siRNA representations into two scores. Finally, another linear SVR model will map the two scores into a final result, as the predicted siRNA efficacy.

## Material and Method

### Datasets

In siRNA researches, Huesken’s dataset is broadly adopted as benchmark, which consists of 2431 siRNA targeted 34 different mRNA. In order to test the machine learning based algorithm, it is commonly divided into a training subset with 2182 siRNA and a testing subset with 249 siRNAs^9^. Another three independent datasets are also accepted to validate the stability of our proposed method in this paper. They include Vicker dataset with 76 siRNAs^18^, Reynolds dataset with 240 siRNAs^7^, and Haborth dataset with 44 siRNAs^19^. Although these datasets provide inhibitions as observed labels, some of them also may be used in classification mode. In such case, 70% targeted gene knockdown is generally considered as the threshold to define active and inactive siRNA.

### Quantitative Representations of siRNA

In this section, we employ several siRNA features formed a representation of siRNA *F*_*Qt*_. These features have one common property: they describe siRNA in quantitative manner. Thereby, the real number values of the features reflect the degree of certain attribute of siRNA. The summary of *F*_*Qt*_ is shown in Table 1.

#### Nucleotide Frequencies

The nucleotide frequencies are the descriptors of nucleotide distribution in siRNA sequence. They were broadly adopted in existed literatures^20^,^21^,^22^. In *F*_*Qt*_, we calculate three groups of nucleotide frequencies by the following rules. The first group indicates the frequencies of A, U, G or C in a siRNA sequence. The second group computes the frequencies of all dinucleotides (e.g., AG, UC, etc), which has 16 possible permutations. The third group represents the frequencies of all trinucleotides (e.g., CAG, UCC, etc), which has 64 possible permutations.

#### Thermodynamic stability profile

The thermodynamic stability is another popular descriptor of siRNA, which demonstrates a guide strand selection mechanism. Many studies had confirmed that the siRNA potency depends strongly on the thermodynamic stability^22^. The thermodynamic stability profile includes Watson-Crick pair free energy Δ*G*, which may be calculated between each two neighboring nucleotides along the siRNA duplex antisense strand in the 5′ to 3′ direction, the sum of all the siRNA local duplex Δ*G*_*duplex*_, and the difference of duplex formation at the 5′ and 3′ end of siRNA for 5 terminal nucleotides ΔΔ*G*. The calculations and results of thermodynamic stability profile may be referred in literatures^23^.

#### Thermodynamic of siRNA-mRNA interaction

Recently, there is mounting evidence that siRNA activity is influenced by the thermodynamic stability of the ends of siRNAs and the energy gain due to hybridization at the siRNA binding site, which determine the accessibility for an interaction between siRNA and mRNA target^24^. Therefore, we would like to include this impact into our predict model, and try to take such thermodynamic parameters into *F*_*Qt*_. To our best knowledge, this is the first work introduces the thermodynamic parameters of siRNA-mRNA binding into siRNA efficacy prediction.

**Figure 1:**
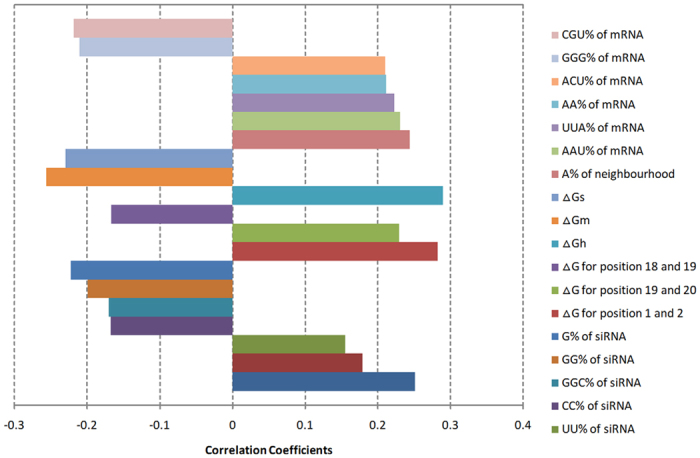
The PCCs between parts of features and siRNA inhibitions on Huesken’s dataset.

The thermodynamic of siRNA-mRNA interaction consists of two components: the energy necessary to make a potential binding region accessible and the energy gained from the base pairing of the two interaction partners^25^. The first component needs two dimensional real numbers to record the free energy for exposing the binding site in siRNA Δ*G*_*s*_ and mRNA Δ*G*_*m*_. The second component describes the energy gained by siRNA-mRNA interaction Δ*G*_*h*_. We can obtain the three thermodynamic parameters using a simple web server tool RNAup developed by Mückstein U in University of Vienna^26^. The tool only needs the sequences of siRNA and targeted mRNA, and will output the three thermodynamic parameters soon. We use RNAup to calculate the thermodynamic parameters of siRNA-mRNA interaction of siRNAs in Huesken’s dataset, and compute their Pearson correlation coefficients (PCC) between the three thermodynamic parameters and observed inhibitions as Fig. 1 shown.

**Figure 2:**
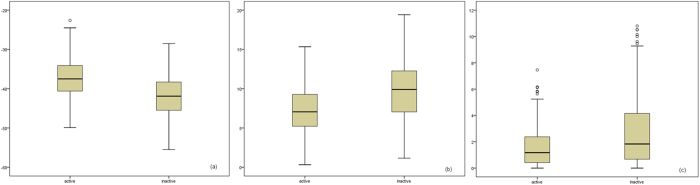
The distributions between active siRNA and inactive siRNA of (**a**) Δ*G*_*h*_ (**b**) Δ*G*_*m*_ (**c**) Δ*G*_*s*_.

In Fig. 1, we also collect the PCCs between some main features in other groups of *F*_*Qt*_ and observed inhibitions. It may be observed that Δ*G*_*h*_ achieves the highest PCC among the three thermodynamic parameters. And the PCCs of three thermodynamic parameters are comparable to those of the features with high PCCs from nucleotide frequencies and thermodynamic stability. Thus they explore the strong correlations between thermodynamic of siRNA-mRNA interaction and siRNA efficacy. Meanwhile, we further investigate their discriminative ability for distinguishing active siRNA from inactive siRNA. We divide siRNAs in Huesken’s dataset into two classes according to the discipline of 70% inhibition of targeted mRNA, and draw the box plots of the three thermodynamic parameters to indicate their distributions between active siRNA and inactive siRNA as Fig. 2.

From Fig. 2, we can observe that the three thermodynamic parameters are discriminative to active and inactive siRNA. Therefore, we believe that they are effective and meaningful for siRNA efficacy prediction.

#### mRNA related features

From the above analyses, we may discover the strong correlations between siRNA efficacy and the thermodynamic parameters of siRNA-mRNA binding. Naturally, we would like to consider using the siRNA-mRNA binding site and corresponding mRNA features for involving more helpful information in *F*_*Qt*_. The literature^15^ shows that less GC content of mRNA at both global and local flanking regions of the siRNA binding sites lead to siRNA inhibition. Inspired by this, we would like to include the mRNA sequence composition and near siRNA binding site into *F*_*Qt*_. We firstly count the frequencies of single-nucleotides, dinucleotides, and trinucleotides in the targeted mRNA sequence, which also have 4, 16, 64 possible permutations respectively. Further, we add up the frequencies of single-nucleotides, dinucleotides, and trinucleotides near siRNA binding site of the targeted mRNA sequence, which also have 4, 16, 64 possible permutations respectively.

### Feature Selection by *F*-score

The above introduced four groups of features are formed a mix feature vector as the quantitative representations *F*_*Qt*_ of siRNA. They quantitatively characterize siRNA from the views of sequence frequencies, thermodynamic stability profile, thermodynamic of siRNA-mRNA interaction and the targeted mRNA. However, because of the lack of direct experimental evidence of these quantitative features linked to siRNA activity, we would like to investigate the contributions among these features in *F*_*Qt*_ by a feature selection method.

*F*-score is a straightforward indicator to measure the discriminative ability of two sets^27^, which is a frequently used feature selection tool for two-class classification problem. The *F*-score of the *i*-th feature can be defined as:


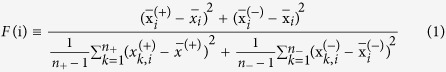


where 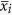, 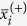, 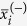 are the average of the *i*-th feature of the whole, positive, and negative samples, respectively. 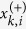 is the *i*-th feature of the *k*-th positive sample, and 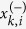 is the *i*-th feature of the *k*-th negative sample. The larger the *F*-score suggests that the involved feature is more discriminative. Therefore it may be a feature selection criterion to select the subset features with more importance. In our algorithm, we label siRNAs in Huesken’s dataset to two categories according to the above mentioned 70% division discipline. Then we calculate the *F*-score of each feature in *F*_*Qt*_ using the simple tool provided by libSVM^28^, and conduct the binary search to choose the best feature subset.


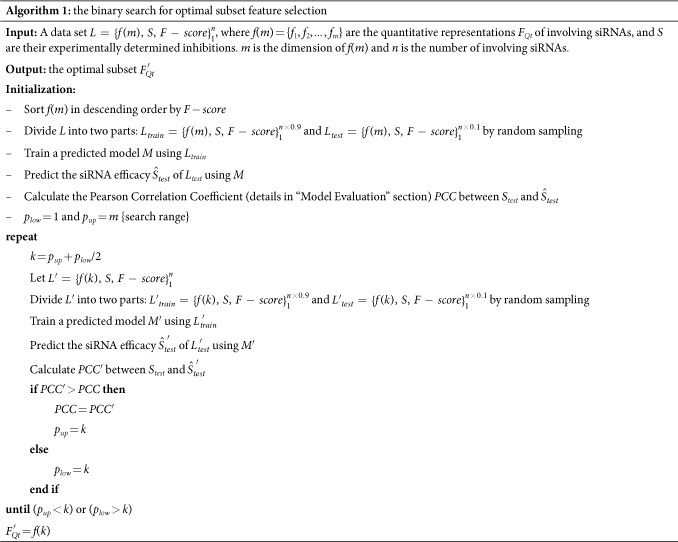


The selective features are deemed strongly relevant to siRNA efficacy, while the absent features are considered weakly relevant. From the experiments (details in “Results of feature selection” section), we obtained 68 dimensional selective features formed the optimal quantitative representations 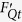.

### Qualitative Representations of siRNA

As previously mentioned, there is another category of important siRNA profiles, i.e empirical rules. The empirical rules experimentally define several patterns regarding siRNA sequence positions for active and inactive siRNA. Differing from *F*_*Qt*_, they are unable to use real number values to accurately describe whether the siRNAs satisfy the rules or not. In this paper, we define another kind of siRNA representations *F*_*Ql*_ using trihedral encoding way (i.e. −1, 0, 1). Because these empirical rules have been validated by biological experiments and analyses, it is unnecessary to conduct feature selection to *F*_*Ql*_. The summary of *F*_*Ql*_ is shown in Table 2.

#### Sequence codes

The siRNA sequence may be seen as the information source for siRNA features. We assign a four dimensional binary code for each nucleotide at sequence. Specifically, the binary coding is A = 〈1, 0, 0, 0〉, C = 〈0, 1, 0, 0〉, G = 〈0, 0, 1, 0〉, U = 〈0, 0, 0, 1〉. The two 3′ overhang nucleotide at position 20 and 21 are also encoded in this features. This encoding way is adopted by several studies^16^,^22^.

#### Rule codes

Several empirical rules suggest that certain nucleotide at certain sequence position may lead to active or inactive siRNA. Such rules for designing siRNA are formulated to a table in literature^16^. In the formulated table, it lists the performance of nucleotide at each position to siRNA efficacy combining 12 rules from the published reports, including Reynolds’s, Ui_tei’s, and Hsieh’s rules^7^,^8^,^10^,^13^,^29^,^30^,^31^,^32^,^33^. We can understand that the nucleotide at each position may prefer for active siRNA or inactive siRNA by seeking the table. Thus we can use the trihedral method to encode each nucleotide at sequence position. The encoding is 1 when the nucleotide prefers for efficient siRNA, while the encoding is −1 when the nucleotide prefers for inefficient siRNA. If no rule mentions such preference, the encoding is 0. However, not all rules provide the preference for all possible nucleotide at a position. In such case, as long as one rule offers a preference suggestion, we will encode the nucleotide at this position by the only rule. For example, if there is an adenine at the seventh position, which satisfies the high-efficacy rule in Svetlana’s, Matveeva’s and Jiang’s rule sets. But other rule sets hardly reveal any preference for adenine at the same seventh position. Therefore, the positional code at seventh position still gets 1 in our works. Further, for a nucleotide at certain position, different rules may possibly explain different preferences. In this paper, we simplify this situation by the principle of majority criterion. For instance, if there is a uracil at the ninth position, which satisfies both the low-efficacy rule in Takasaki’s rule set and the high-efficacy rule in Svetlana’s and Jiang’s rule set. Under this circumstance, we will adopt the positional code at ninth position as 1, because more rules support this kind of preference. In light of out simplified approach, the table of preference for nucleotides at each position from literature^16^ may be re-formulated as Table 3 shown. Thereby, one can rapidly find out the encoding for nucleotides at each position.

### Multiple representations fusion model based on SVR at score level

Next, we would like to propose a fusion model for combining the selective quantitative representation 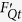 and qualitative representations *F*_*Ql*_ of siRNA at score level. The key of this model is to use Supported Vector Regression (SVR) with regard to the two kinds of siRNA representations. The SVR is an effective and widely applicable regression tool^34^. The idea of SVR is based on the computation of a regression function in a high-dimensional feature space where the input data are mapped via a linear or nonlinear function. Its regression function is defined as follows:


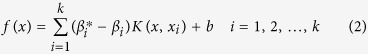


where *k* is the number of training data. The Lagrangian multipliers 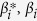 are found by solving a quadratic programming problem^35^. And *b* is the bias. The kernel function performs a linear or non-linear mapping, which can employ any symmetric function satisfied Mercer’s condition. The most widely used kernels include linear, polynomial, radial basis function (RBF), and sigmoid kernel^36^, which extend SVR’s ability to handle all types of data.

In our model, the first stage is to model two SVRs with reasonable kernels for distinctively mapping the two kinds of siRNA representations 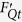 and *F*_*Ql*_ to their corresponding predicted scores. By our traversing experiments (details in “Performance of two representations and their fusion” section), the linear-SVR and RBF-SVR are more appropriate with regard to 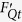 and *F*_*Ql*_ respectively. The two estimated scores independently represent the predicted activities by the single siRNA representation 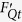 and *F*_*Ql*_. In the second stage, the remaining problem is transformed to find another regression function using the two estimated scores as input. We thus train another linear-SVR model to map the two scores into a final result. This final label may be seen as the predicted siRNA efficacy by fusing multiple the siRNA representations 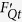 and *F*_*Ql*_ for consolidating the siRNA efficacy prediction. In summary, Algorithm 2 formulizes the steps described above.


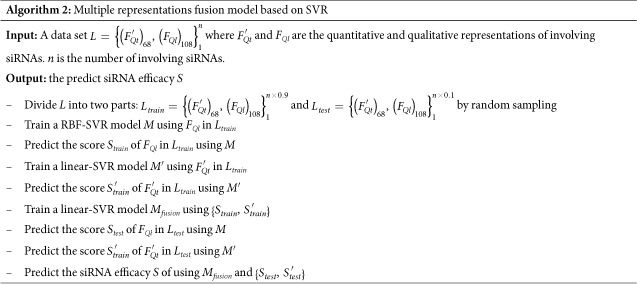


### Model Evaluation

In this article, we adopt Pearson Correlation Coefficient (PCC) to measure the correlation between the predicted efficacy and observed inhibitions, which is the most common use in a regression system. Its definition is as follow:


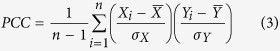


Where *X* and *Y* represents the predicted values and observed labels. *n* is their common size. 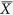 and *σ*_*X*_ denote the mean and standard deviation of *X* respectively. Likewise, *Y* and *σ*_*Y*_ denote the mean and standard deviation of *Y* respectively.

As above mentioned, some literatures also conducted the experiments of predicting siRNA efficacy in classification way. Therefore, some classification indicators, including sensitivity and specificity are also employed in our work. These indicators can be calculated as follows:


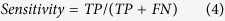



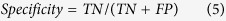


Where *TN, FN, TP* and *FP* are the number of true negatives, false negatives, true positives and false positives respectively.

In addition, the Receiver Operating Characteristic (ROC) curve is also used to exhibit the overall performance of algorithms. The ROC curve is drawn by plotting the true positive rate (i.e. sensitivity) versus the false positive rate (i.e. 1 – specificity) with different thresholds. In ROC, we may further observe the area under ROC curve (AUC) to evaluate the reliability of classification system. A perfect classification system may obtain the maximum AUC value 1, while the AUC value 0.5 implies a random classification.

## Results

### Results of feature selection

We would like to report the details of feature selection for *F*_*Qt*_ first. We respectively calculate the *F*-scores of 275 features in *F*_*Qt*_ according to section 2.3, and employ binary search strategy to find the optimal subset features by the descending sorted 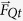. The Table 4 shows the processes of binary search for the optimal subset features 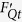.

**Figure 3:**
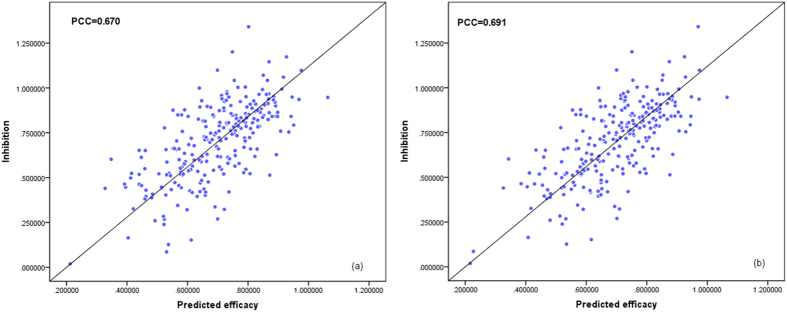
The comparisons between two linear-SVR models using (**a**) *F*_*Qt*_ and (**b**) 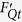.

In Table 3, we firstly use all 275 features 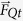 to train a SVR model with linear kernel on Huesken_train dataset, and then test the regression model on Huesken_test dataset. Although the PCC of 275 features has achieved 0.670, we need to continuously try the half part of 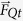. Such an attempt will go on until the PCC drops for the first time at the fourth iteration. At that time, we will try to obtain the optimal feature subset between 34 dimensional subset of 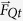 and 68 dimensional subset of 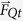. The binary search continues until it can reach an optimal subset of 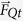 with a higher PCC than 0.691. After the whole searching, we get the 68 dimensional subset of 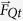 with the highest PCC 0.691 as selected representation 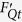. The comparisons between two linear-SVR models using *F*_*Qt*_ and 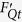 are shown as Fig. 3.

**Figure 4:**
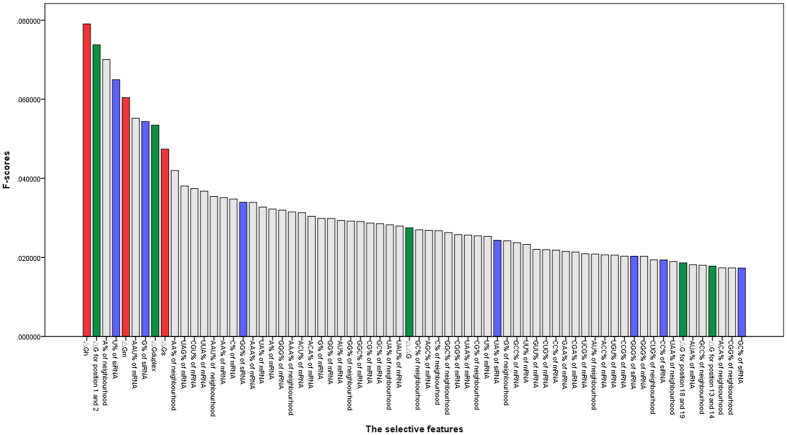
The 68 dimensional selective features by *F*-scores.

We also exhibit the 68 selective features in 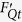 as Fig. 4 shown. In Fig. 4, the selective features are listed descending order by *F*-scores. We can note that the selective features are from all four groups, where our proposed the thermodynamic parameters of siRNA-mRNA interaction Δ*G*_*h*_, Δ*G*_*m*_ and Δ*G*_*s*_ rank the first, the fifth and the ninth according to their *F*-scores. Their highest 100% selected rate demonstrates such category of features may provide significant contributions to siRNA efficacy prediction.

In the group of mRNA related features, 53 features are selected: A% of neighbourhood, AAU% of mRNA, AA% of neighbourhood, UAG% of mRNA, CGU% of mRNA, UUA% of mRNA, AAU% of neighbourhood, AA% of mRNA, C% of mRNA, AAA% of mRNA, UA% of mRNA, A% of mRNA, GGG% of mRNA, AAA% of neighbourhood, ACU% of mRNA, ACA% of mRNA, G% of mRNA, GG% of mRNA, AU% of mRNA, GG% of neighbourhood, GGC% of mRNA, CG% of mRNA, GC% of mRNA, UA% of neighbourhood, UAU% of mRNA, GC% of neighbourhood, AGC% of mRNA, C% of neighbourhood, GGC% of neighbourhood, CGG% of mRNA, UAA% of mRNA, CG% of neighbourhood, U% of mRNA, G% of neighbourhood, GCC% of mRNA, UU% of mRNA, GUU% of mRNA, CUG% of mRNA, CC% of mRNA, GAA% of mRNA, CGA% of mRNA, UCG% of mRNA, AU% of neighbourhood, ACC% of mRNA, UGU% of mRNA, CCG% of mRNA, GGG% of mRNA, CUG% of neighbourhood, UAA% of neighbourhood, AUA% of mRNA, GCC% of neighbourhood, ACA% of neighbourhood, and CGG% of neighbourhood. Such a large quantity of selective features and high selective rate indicate that the mRNA related features needs to be part of siRNA representation.

In the group of thermodynamic stability profile, five features are selected: Δ*G* for position 1 and 2, Δ*G*_*duplex*_, ΔΔ*G*, Δ*G* for position 18 and 19, Δ*G* for position 13 and 14. Their 25% selective rate and high *F*-scores show that such category of features may help to improve siRNA efficacy prediction.

In the group of nucleotide frequencies, seven features are selected: U%, G%, GG%, UA%, GGG%, CC% and GC% of siRNA in the order. Their 8.33% selective rate exhibits that only a small number of them have strong correlation to siRNA efficacy prediction. But the above selective features imply that the content of G/GC/UA in siRNA sequence should be considered as important siRNA design rules, which are consistent with the conclusions of Reynolds and Tei^7^,^8^.

### Performance of two representations and their fusion

After obtaining the selective quantitative representation 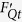, we may separately create two SVR models for mapping the two categories of siRNA representations 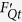 and *F*_*Ql*_ into two sets of predicted scores on Hencken_train dataset. Further, let 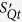 and S_Ql_ as the two sets of scores from Hencken_train dataset, and they are arranged to train another SVR model to produce the final predicted results. We train these SVR models with 10-fold cross validation using the libSVM tool^28^, and then test the trained model using the siRNAs in Hencken_test dataset.

In order to construct rational SVR models, we attempt to separately traverse 4 popular SVR kernels for the single siRNA representations 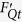 and *F*_*Ql*_, and the predicted scores 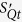 and *S*_*Ql*_ as inputs. Furthermore, we also perform the way of combining the 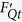 and *F*_*Ql*_ into a feature vector *F*_*Ql*+*Qt*_ using the same experimental protocol for comparisons. The combined vectors *F*_*Ql*+*Qt*_ with 171(=68 + 103) dimensional real and discrete components of siRNAs in Hencken_train dataset are used to train SVR models and traverse the four kernels. The Table 5 shows the PCCs produced by these SVR models on Hencken_test dataset.

**Figure 5:**
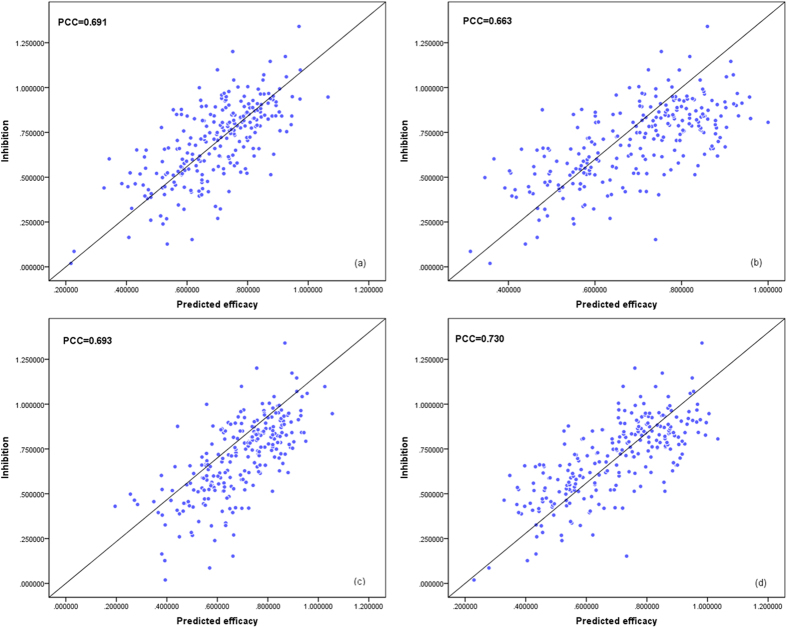
The predicted results from the models for (**a**) 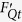 (**b**) *F*_*Ql*_ (**c**) *F*_*Ql*+*Qt*_ and (**d**) our proposed fusion method.

In Table 5, the best performed kernels regarding different siRNA representations and inputs are diverse. For 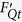, the highest PCC emerges when SVR using linear kernel, while the excellent performance of *F*_*Ql*_ is achieved by RBF kernel. We believe that the difference comes from their different data types. The phenomenon also prompts us that it is not so reasonable to combine these fundamental different representations into one feature vector. Putting the PCCs of the experiment using 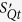 and *S*_*Ql*_ and the experiment using *F*_*Ql*+*Qt*_ together, we may note that the best PCC among four kernels using *F*_*Ql*+*Qt*_ as input is 0.693, which is 5.3% lower than our score level fusion method. When we train the SVR model for fusing the two predicted scores 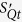 and *S*_*Ql*_, the linear-SVR model acts the outperformance. It demonstrates that the predicted scores 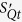 and *S*_*Ql*_ are prone to a simple linear combination way due to their homogeneity. The predicted results from the models for 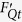, *F*_*Ql*_, *F*_*Ql*+*Qt*_ and our proposed fusion method are shown in Fig. 5. From these figures, we can conclude that our score level fusion algorithm may take advantage of the two kinds of siRNA representations, and achieve better performance than the model with only single siRNA representation. Moreover, it can be considered a more rational combination approach for multiple siRNA features than the popular way of forming multiple features as an input vector.

### Comparisons of algorithms

**Figure 6:**
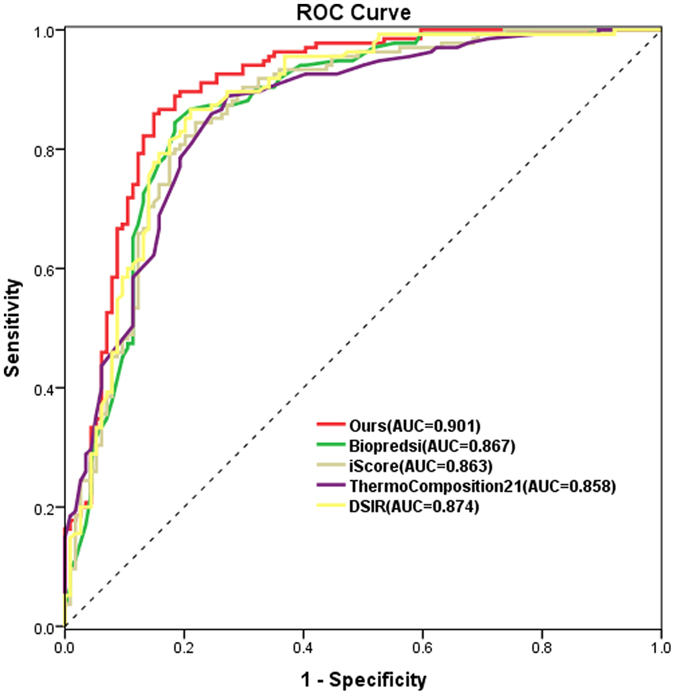
The ROC curves of the five algorithms.

In order to further exhibit the advantage of our proposed methods, we conduct a serial of comparative experiments among our approaches and the most state-of-the-art systems Biopredsi^9^, ThermoComposition-21^10^, DSIR^11^ and i-score^12^ both in the classification and regression modes. The 70% threshold of targeted gene knockdown is also used to separate active and inactive siRNAs in Hencken dataset. All models of these methods are trained on Hencken_train dataset and tested on Hencken_test dataset. The ROC curves with sensitivity, specificity and AUC of our method and the four systems are plotted in Fig. 6. In Fig. 6, we may discover that our method the highest ROC curve and the best AUC of 0.901 perform among the comparative five algorithms. Table 6 details the performance of our method and the four systems. As Table 6 shown, the PCC of our method achieves 0.730, which is 10.61%, 11.62%, 10.77% and 8.96% higher than the algorithms of Biopredsi, i-score, ThermoComposition-21 and DSIR respectively. In siRNA design, false positives prediction will take more experimental cost, thus siRNA design tools are expected to be capable of controlling false positives (high specificity) and retaining the maximum number of true positives (high sensitivity). In order to exhibit such requirements, Table 6 also compares three groups of sensitivities together with high specificities 90.7%, 96.5% and 99% for each algorithm. In these groups, our model may achieve highest sensitivities among all the algorithms, when the specificities get high. It well indicates the high confidence of our algorithm.

**Figure 7:**
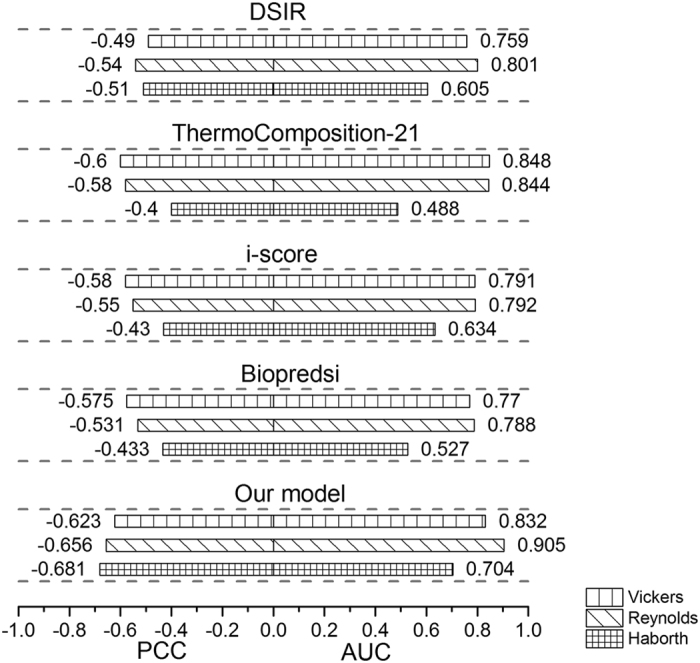
The comparisons of five algorithms testing on the three independent datasets of Vickers, Reynolds and Harborth.

For testing the stability of our method, we conducted extensive comparative experiment among the five algorithms. In these experiments, the models of the five algorithms are trained on Hencken_train dataset but tested on the three independent datasets of Vickers, Reynolds and Harborth. We collect the PCCs and AUCs generated from the experiments in Fig. 7.

In Fig. 7, it shows that our method also can achieve the highest PCCs compared with other four algorithms on all three independent testing datasets and obtained higher AUCs except when tested on Vickers’ dataset. Otherwise, our method may produce more stable results across each of the independent siRNA datasets. In summary, our method outperforms other four algorithms in term of effectiveness and stability in all comparative experiments. We believe that such improvement is ascribed to the synthetical process of the thermodynamic of siRNA-mRNA interaction, targeted mRNA, our feature selection method and the multiple representation fusion at score level.

## Conclusion

In this article, we present a siRNA efficacy prediction method by combining two kinds of siRNA representations at score level. We first introduce the thermodynamic of siRNA-mRNA interaction together with nucleotide frequencies, the thermodynamic stability profile, and mRNA-related features as a 275 dimensional siRNA quantitative representation. Further, we adopt *F*-score as an importance measure to evaluate all features in such siRNA quantitative representation. The top-ranked 68 dimensional features are chosen, which performs highest *F*-scores among all possible feature subsets. Our proposed thermodynamic parameters of siRNA-mRNA interaction are 100% included in selective features with high *F*-scores, which suggests that such category of features may provide significant contributions to siRNA activity prediction. We also find that the features selected from nucleotide frequencies are consistent with the design rules from the researches of Reynolds and Tei. In addition, we also encode siRNA sequence and several empirical rules as the qualitative representations of siRNA. In order to maximize the strengths of both quantitative and qualitative representations of siRNA, we trained a fusion model based on SVR for combining the two kinds of representations at score level. The experimental data validate the outperformance of our model. Even in the extensive experiments on the independent datasets of Vickers, Reynolds and Harborth, our method also show more stability and better performance than several popular siRNA efficacy prediction systems.

## Additional Information

**How to cite this article:** He, F. *et al*. Predicting siRNA efficacy based on multiple selective siRNA representations and their combination at score level. *Sci. Rep.* **7**, 44836; doi: 10.1038/srep44836 (2017).

**Publisher's note:** Springer Nature remains neutral with regard to jurisdictional claims in published maps and institutional affiliations.

## Figures and Tables

**Table 1:** The brief introduction of *F*_*Qt*_.

Group	Feature	Dimension
Nucleotide frequencies	Single-nucleotide frequencies	4
	Dinucleotide frequencies	16
	Trinucleotide frequencies	64
Thermodynamic stability profile	Watson-Crick pair free energy	18
	The sum of all the siRNA local duplex	1
	The difference of duplex formation at the 5′ and 3′ end of siRNA for 5 terminal nucleotides.	1
Thermodynamic of siRNA-mRNA interaction	the energy necessary to make a potential binding region accessible	2
	the energy gained from siRNA-mRNA interaction	1
mRNA related features	Single-nucleotide frequencies in mRNA	4
	Dinucleotide frequencies in mRNA	16
	Trinucleotide frequencies in mRNA	64
	Single-nucleotide frequencies in near siRNA binding site region of mRNA	4
	Dinucleotide frequencies in near siRNA binding site region of mRNA	16
	Trinucleotide frequencies in near siRNA binding site region of mRNA	64

**Table 2:** The brief introduction of *F*_*Ql*_.

group	Encoding rule	Dimension of features
Sequence codes	Map nucleotides at each sequence position to four dimensions in vector space	84
Rule codes	Encode nucleotides at each sequence position with rule sets	19

**Table 3:** The encoding for nucleotide at each position in light of empirical rules.

Position	Nucleotide	Encoding	Rule providers
1	A	−1	Ui-Tei, Amarzguioui, Takasaki, Svetlana, Matveeva
	C	+1	Ui-Tei, Amarzguioui, Jagla 1, Jagla 2, Jagla 3, Matveeva
	G	+1	Ui-Tei, Amarzguioui, Takasaki, Svetlana, Jagla 1, Jagla 2, Jagla 3, Matveeva, Jiang
	U	−1	Ui-Tei, Amarzguioui, Takasaki, Svetlana, Matveeva, Jiang
2	A	−1	Amarzguioui
	C	0	
	G	+1	Svetlana, Jiang
	U	−1	Amarzguioui, Matveeva
3	A	+1	Reynolds
	C	−1	Matveeva
	G	+1	Svetlana, Jiang
	U	−1	Amarzguioui, Svetlana, Jiang
4	A	0	
	C	−1	Svetlana
	G	0	
	U	+1	Matveeva
5	A	+1	Jagla 4
	C	0	
	G	0	
	U	+1	Jagla 4
6	A	+1	Amarzguioui, Takasaki, Svetlana, Jagla 4, Matveeva, Jiang
	C	−1	Hsieh, Takasaki, Svetlana, Matveeva, Jiang
	G	−1	Svetlana, Svetlana
	U	+1	Svetlana, Jagla 4, Matveeva, Jiang
7	A	+1	Svetlana, Matveeva, Jiang
	C	−1	Svetlana, Matveeva, Jiang
	G	+1	Takasaki
	U	−1	Takasaki
8	A	+1	Takasaki
	C	0	
	G	−1	Takasaki
	U	0	
9	A	0	
	C	0	
	G	−1	Takasaki, Matveeva
	U	−1	Jagla 1, Jiang
10	A	+1	Jagla 1
	C	+1	Jagla 2
	G	+1	Jagla 2
	U	+1	Reynolds, Svetlana, Jagla 1, Matveeva, Jiang
11	A	0	
	C	+1	Hsieh, Jagla 3
	G	+1	Hsieh, Jagla 3
	U	0	
12	A	+1	Matveeva
	C	0	
	G	−1	Matveeva
	U	0	
13	A	+1	Svetlana, Matveeva, Jiang
	C	−1	Svetlana, Jiang
	G	−1	Reynolds, Svetlana, Jiang
	U	+1	Svetlana, Matveeva, Jiang
14	A	0	
	C	−1	Svetlana, Jiang
	G	0	
	U	0	
15	A	+1	Svetlana, Jiang
	C	−1	Matveeva
	G	0	
	U	−1	Svetlana, Jiang
16	A	0	
	C	0	
	G	+1	Hsieh
	U	+1	Matveeva
17	A	+1	Amarzguioui, Svetlana, Matveeva, Jiang
	C	0	
	G	−1	Matveeva
	U	+1	Amarzguioui
18	A	+1	Amarzguioui, Svetlana, Matveeva, Jiang
	C	−1	Svetlana, Matveeva, Jiang
	G	−1	Matveeva
	U	+1	Svetlana
19	A	+1	Ui-Tei, Amarzguioui, Svetlana, Jagla 1, Jagla 2, Jagla 4, Matveeva, Jiang
	C	−1	Reynolds, Ui-Tei, Matveeva, Jiang
	G	−1	Reynolds, Ui-Tei, Amarzguioui, Hsieh, Takasaki, Svetlana, Matveeva, Jiang
	U	+1	Ui-Tei, Amarzguioui, Hsieh, Svetlana, Jagla 1, Jagla 2, Jagla 4, Matveeva, Jiang

+1: Preference for high siRNA efficacy. −1: Preference for low siRNA efficacy. 0: No rule followed.

**Table 4:** The processes of binary search for the optimal subset features 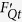 .

Iteration	Number of features	Pearson Correlation Coefficient
1	275	0.670
2	275/2 = 137	0.682
**3**	**137**/**2 = 68**	**0.691**
4	68/2 = 34	0.684
5	34 + (68–34)/2 = 51	0.688
6	51 + (68–51)/2 = 59	0.687
7	59 + (68–59)/2 = 63	0.685
8	63 + (68–63)/2 = 65	0.684
9	65 + (68–65)/2 = 66	0.687

**Table 5:** The PCCs produced by the SVR models with different kernels and different inputs on Hencken_test dataset.

Input	PCC			
	Linear	polynomial	RBF	sigmoid
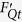	**0.691**	0.613	0.401	0.017
*F*_*Ql*_	0.430	0.589	**0.663**	0.366
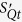 and *S*_*Ql*_	**0.730**	0.667	0.697	0.007
*F*_*Ql*+*Qt*_	0.577	0.454	**0.693**	0.002

**Table 6:** The details of performance of the five algorithms.

Method	PCC	AUC	Sensitivity	Specificity
Biopredsi	0.660	0.867	45.2%	90.7%
			17%	96.9%
			9.6%	99.0%
i-score	0.654	0.863	48.1%	90.7%
			24.4%	96.9%
			8.9%	99.0%
ThermoComposition-21	0.659	0.858	50.4%	90.7%
			28.9%	96.9%
			16.5%	99.0%
DSIR	0.670	0.874	58.5%	90.7%
			25.9%	96.9%
			14.8%	99.0%
**Ours**	**0.730**	**0.901**	**67.4%**	**90.7%**
			**20.7%**	**96.9%**
			**17.8%**	**99.0%**
